# Comparison of Parent Questionnaires, Examiner-Led Assessment and Parents’ Concerns at 14 Months of Age as Indicators of Later Diagnosis of Autism

**DOI:** 10.1007/s10803-019-04335-z

**Published:** 2019-12-16

**Authors:** Greg Pasco, Kim Davies, Helena Ribeiro, Leslie Tucker, Carrie Allison, Simon Baron-Cohen, Mark H. Johnson, Tony Charman, The BASIS Team, Rachael Bedford, Anna Blasi, Patrick Bolton, Celeste Cheung, Mayada Elsabbagh, Janice Fernandes, Isobel Gammer, Natasa Ganea, Teea Gliga, Jeanne Guiraud, Ute Liersch, Michelle Liew, Sarah Lloyd-Fox, Helen Maris, Louise O’Hara, Andrew Pickles, Erica Salomone

**Affiliations:** 1 Department of Psychology, King’s College London, Institute of Psychiatry, Psychology and Neuroscience, Henry Wellcome Building, 16 De Crespigny Park, London, SE5 8AF UK; 2 Centre for Brain and Cognitive Development, Birkbeck College, University of London, London, UK; 3 Department of Psychiatry, Autism Research Centre, University of Cambridge, Cambridge, UK; 4 Department of Psychology, University of Cambridge, Cambridge, UK

**Keywords:** Autism, Early detection, Behavioural signs, Assessment, Infant siblings

## Abstract

Parents participating in a prospective longitudinal study of infants with older siblings with autism completed an autism screening questionnaire and were asked about any concerns relating to their child’s development, and children were administered an interactive assessment conducted by a researcher at 14 months. Scores on the parent questionnaire were highest for children later diagnosed with autism. Parental concerns and scores from the examiner-led assessment distinguished children with later developmental difficulties (both autism and other developmental atypicalities) from those who were developing typically. Children about whom parents expressed concern scored higher on both the questionnaire and the interactive assessment than those without concerns. There were no significant associations between total or individual item scores from the questionnaire and interactive assessment.

**Electronic supplementary material:**

The online version of this article (10.1007/s10803-019-04335-z) contains supplementary material, which is available to authorized users.

The development of effective instruments to screen for autism in toddlers has been a long-standing aim for researchers and clinicians alike, with the expectation that early identification will lead to earlier diagnosis and intervention, reduce stress and anxiety for parents and promote better outcomes for young children on the autism spectrum (Zwaigenbaum et al. 2013). However, attempts to design valid and reliable early parent-report screening tools for autism have had mixed results, with conflicting views about the value of their routine use in a population-wide context. Some official guidelines recommend early screening for autism in primary care settings (Johnson et al. 2007; Volkmar et al. 2014), whereas others suggest that existing screening instruments lack sufficient qualities to justify their use in community-wide early years services (e.g. American Academy of Family Physicians 2016; Siu et al. 2016; UK National Screening Committee 2012). When used at around 18 months of age screening instruments may be less effective at detecting children who go on to be diagnosed with autism than when used with older toddlers, and different aspects of development have been shown to be informative at different ages (Stenberg et al. 2014; Sturner et al. 2017a, b; Toh et al. 2017). Sacrey et al. (2018) describe the range of currently available screeners for autism in pre-schoolers for which evaluations have been conducted.

In addition to the use of screening questionnaires, parents of young children may express explicit concerns about their child’s early development. Information about the focus of these concerns in relation to children later diagnosed with autism is often based on retrospective recall following diagnosis. One large-scale study (Zuckerman et al. 2015) found that parents of children later diagnosed with ASD reported first concerns about their child’s development at approximately 24 months of age, almost a year earlier than parents of children with either developmental delay or learning disability. However, parents of the children later diagnosed with autism were more likely to receive a reassuring, passive response from professionals than the more proactive responses relating to the children with non-autistic disabilities.

Prospective longitudinal studies involving young children at elevated likelihood (EL)^1^ of autism due to having an older sibling with an autism diagnosis provide an opportunity to elicit and investigate parental concerns relating to children’s very early development. Sacrey et al. (2015) used a semi-structured interview to explore parents’ concerns up to six times between 6 and 24 months of age. Concerns about sensory and motor skills predicted autism diagnostic outcome as early as 6 months, and concerns about sensory and play skills by 9 months. Concerns about social development did not predict outcome until 12 months, communication not until 15 months and repetitive behaviours and restricted interests not until 18 months.

Studies report that stereotyped and repetitive behaviour elicited in examiner-led interactive assessments may indicate risk for later autism diagnosis from as early as 12 months of age (e.g. Elison et al. 2014). Ozonoff et al. (2010) suggest that differences between children later diagnosed with autism and their typically developing counterparts may be evident from 12 months of age, based on observational measures of early social behaviour. Chawarska et al. (2014) investigated the features of early social communication derived from the Autism Diagnostic Observation Schedule-Generic (ADOS-G: Lord et al. 2000), a semi-structured observational measure of autism symptomatology, administered at 18 months. In this prospective longitudinal study of siblings at EL of autism, different profiles of difficulties and behaviour predicted later diagnosis of ASD, including: poor eye contact plus lack of communicative gestures and giving; poor eye contact with a lack of imaginative play; and repetitive behaviours in combination with a lack of gestures.

Sacrey et al. (2018) compared items from the Autism Parent Screen for Infants (APSI: Sacrey et al. 2018), a 26-item parent-report questionnaire, with corresponding items from the Autism Observation Scale for Infants (AOSI: Bryson et al. 2008), a semi-structured examiner-led assessment designed to detect and monitor signs of emerging autism traits. There was poor agreement between 19 matched items across the two instruments, and items from the parent report at both 12 months and 18 months were generally better than the examiner-based measure in predicting later autism diagnosis. AOSI total score has been shown to discriminate later ASD outcome at 14 months in another elevated likelihood sibling study (Gammer et al. 2015) and scores from 9 AOSI items at 18 months contributed to the prediction of later ASD diagnosis in the longitudinal prospective sibling study reported by Brian et al. (2008).

The Quantitative Checklist for Autism in Toddlers (Q-CHAT: Allison et al. 2008) is a normally-distributed 25-item questionnaire designed to screen for autism in toddlers in the general population. The utility of the Q-CHAT as a primary screening tool for autism has yet to be demonstrated, although a 10-item version of the Q-CHAT has been reported to show potential utility in highlighting early ‘red flags’ for autism (Allison et al. 2012) and Raza et al. (2019) report that this shorter version can predict later ASD diagnosis at 18 and 24 months of age in an EL study context.

The present study involves participants who were recruited as part of a prospective longitudinal study of children at elevated likelihood of autism due to having older siblings with a diagnosis of autism. Children recruited for such studies are likely to display fewer and less severe symptoms than those recruited on the basis of clinical referral or existing diagnoses (Sacrey et al. 2017) and instruments whose properties may provide greater variance across this type of sample may be more informative than those that have less normally-distributed features. For this reason, the Q-CHAT was deemed to be appropriate as a parent-report measure of autism symptomatology. We investigated scores from Q-CHAT and AOSI assessments and also measured the expression of explicit parental concerns about their child’s development at 14 months. We predicted that Q-CHAT and AOSI total scores and the expression of parental concerns would relate to autism diagnosis at 3 years of age. We also investigated the additional value of combining results from these three sources of information.

## Method

### Participant Recruitment and Assessment Visits

Children participating in the British Autism Study of Infant Siblings (BASIS: http://www.basisnetwork.org/) were assessed at approximately 8 months, and then at 14, 25 and 38 months. Participants comprised 113 children (63 male; 50 female) with older siblings with autism (Elevated Likelihood: EL) and 27 children (14 male; 13 female) with at least one older sibling and no history of autism in first-degree relatives (Typical Likelihood: TL). Three additional EL children were not seen at either the 25- and 38-month visits and were excluded from all analyses presented here. TL children were born full-term (37–42 weeks), except one born prior to 37 weeks, and were recruited via a volunteer database held at the Birkbeck College Centre for Brain and Cognitive Development. Full details of EL and TL older sibling diagnostic status are shown in the Supplementary Materials. Participants were administered a range of experimental, behavioural and cognitive tasks and interactive assessments of autistic symptomatology. Parents completed a range of questionnaires and interviews relating to different aspects of early development.

### Measures

Cognitive ability was assessed at each visit using the Mullen Scales of Early Learning (MSEL: Mullen 1995), a standardised measure of early nonverbal reasoning, motor and language skills. At 25 and 38 months autism symptomatology was assessed using the ADOS-G. At the 3-year visit parents were interviewed using the Autism Diagnostic Interview-Revised (ADI-R: Lord et al. 1994), a detailed interview covering early development and autism diagnostic features. The Q-CHAT is a 25-item questionnaire with items relating to early social communication and play skills, and restricted, repetitive, stereotyped and sensory behaviours. Each item has 5 options, based on relative frequency, intensity or typicality, and is scored 0 to 4, with higher scores relating to increased putative autism severity. All item scores are summed to provide a total score. Parents were asked to complete the Q-CHAT prior to the 14-, 25- and 38-month visits. The AOSI is an examiner-led interactive assessment with an infant, who is usually sitting on a caregiver’s lap. Tasks designed to elicit early social communication and play behaviours are administered, and 19 items relating to autistic symptomatology are scored from 0 to 2 or 3, with higher scores relating to increased putative autism severity. Sixteen item scores are summed to provide a total score. The AOSI was administered at the 8- and 14-month visits. At each visit parents were asked about any concerns relating to the child’s health or development.

### Participant Diagnostic Classification

Following the final visit experienced researchers (GP, TC) and members of the testing team assigned a best estimate research diagnosis of DSM-5 autism (EL-Autism) or non-autism to each EL child, informed by, but not dependent on, outcomes from the ADOS-G, ADI-R and Mullen assessments as well as researcher observations from the assessment visits at 2 and 3 years of age. Non-autistic EL children were classified as typically developing (EL-TD) or ‘other’ (EL-Other). Children were assigned to the EL-Other group if they met the following criteria at the 3-year visit: scoring above the autism spectrum threshold on the ADOS-G, and/or scoring above the autism spectrum (Risi et al. 2006) threshold on the ADI-R, and/or scoring below – 1.5 SD on one or more of the Mullen *Early Learning Composite*, *Visual Reception*, *Receptive Language* or *Expressive Language* subscales.

### Classifying Parental Concerns About Their Children’s Development

At each visit parents were asked whether they had any concerns about their child’s development. Any concerns expressed were recorded verbatim by a researcher. Responses were later classified independently by two raters, blind to group status, in relation to eleven pre-specified categories: *No concerns*; *Medical* (including allergies and asthma); *Sleep*; *Language* (delayed, unusual, stereotyped); *Social* (relating to children and/or adults); *Stereotyped behaviour* (repetitive, rigid, unusual); *Behaviour* (overactive, problem); *Motor* (delayed, unusual); *Sensory* (interests, aversion); *Autism spectrum*; *Other/General*. Raters assigned a single category most representative of the description given. For the 47 cases where at least one rater coded a concern, inter-rater agreement for classification was moderate (Cohen’s *κ *= 0.572, *p *< .001). Inter-rater agreement between each of the raters and GP’s independent ratings were higher (*κ *= 0.755, *N *= 44 & *κ *= 0.717, *N *= 46, both *p *< .001). Discrepancies between the two blinded raters were resolved by adopting the category assigned independently by GP. For the purpose of this analysis, the *Medical*, *Sleep* and *Other/General* categories—representing 6, 2, and 1 cases respectively—were equated to having no concerns.

### Intervention Trial Supplementary Analysis

Fifty-three of the EL participants in this sample took part in a randomized controlled trial (RCT) of a parent-mediated early intervention programme (Green et al. 2017), with the active treatment period between the 8- and 14-month visits. Twenty-seven children were in the intervention arm of the trial, and a further five children took part in a case series pilot of the intervention (Green et al. 2013). To investigate whether enrolment in the RCT or receiving the intervention affected the main outcomes described above for each of the three measures we conducted several supplementary analyses.

## Results

### Diagnostic Outcomes and Participant Characterisation

Following the 3-year visit 17 EL children were identified as having autism (EL-Autism; 15 males: 2 females) as per the procedure described in the Method section above. Sixty-four EL children were considered to be typically developing (EL-TD; 28 males: 36 females); 32 non-autistic EL children were classified as not developing typically (EL-Other; 20 males: 12 females). Of the children in the EL-Other group, 12 met ADOS-G criteria only, 3 met both ADOS-G and ADI-R criteria, 1 met ADI-R criteria only, 5 met both ADOS-G and MSEL criteria, 10 met MSEL criteria only and 1 met ADOS-G, ADI-R and Mullen criteria. Details of age, MSEL, AOSI, ADOS and ADI-R scores at each visit are shown in Table 1. Missing data for Q-CHAT Total, AOSI Total, MSEL ELC, ADOS-2 CSS and ADI-R Toddler Total scores and ages at assessment were imputed using expectation maximisation procedures in SPSS version 25.

**Table 1:** Age, mullen early learning composite, autism diagnostic observation schedule and autism diagnostic interview—revised scores at 14 and 38 months: mean (SD) and between-group differences

	TL^1^ (*n *= 27)	EL-TD^2^ (*n *= 64)	EL-Other^3^ (*n *= 32)	EL-Aut^4^ (*n *= 17)	
Age at 14-month visit	15.0 (0.90)	14.8 (0.94)	14.9 (0.95)	14.8 (0.95)	*F*(3,136) = 0.417, *p *= .741, partial *η*^2^ = 0.009
Age at 38-month visit	38.7 (1.56)	38.9 (1.44)	38.7 (1.87)	38.6 (1.66)	*F*(3,136) = 0.299, *p *= .826, partial *η*^2^ = 0.007
Mullen ELC* at 14 months	102.6^a,b^ (14.43)	98.2^c^ (12.48)	91.5^a^ (15.84)	85.5^b,c^ (12.81)	*F*(3,136) = 7.053, *p *< .001, partial *η*^2^ = 0.135
Mullen ELC* at 38 months	118.5^a,b^ (15.13)	114.4^c,d^ (15.80)	87.4^a,c^ (25.59)	86.0^b,d^ (27.77)	*F*(3,136) = 22.130, *p *< .001, partial *η*^2^ = 0.328
ADOS-2 CSS^§^ at 38 months	2.0^a,b^ (1.54)	1.3^c,d^ (0.54)	4.1^a,c^ (2.31)	3.8^b,d^ (3.32)	*F*(3,136) = 22.24, *p *< .001, partial *η*^2^ = 0.329
ADI-R^†^ Toddler Total at 38 months	1.0^a,b^ (1.38)	1.8^c,d^ (2.27)	4.0^a,c,e^ (5.26)	16.2^b,d,e^ (6.65)	*F*(3,136) = 72.522, *p *< .001, partial *η*^2^ = 0.615

Superscript letters indicate between-group differences at *p *< .05

^1^Typical Likelihood

^2^Elevated Likelihood—typical development

^3^Elevated Likelihood—other

^4^Elevated Likelihood—autism

*Early learning composite

^§^Autism Diagnostic Observation Schedule—Second edition Calibrated Severity Score (NB ADOS-2 CSS scores were calculated using ADOS-G item scores)

^†^Autism Diagnostic Interview—revised

### Q-CHAT Scores at 14 Months

**Fig. 1:**
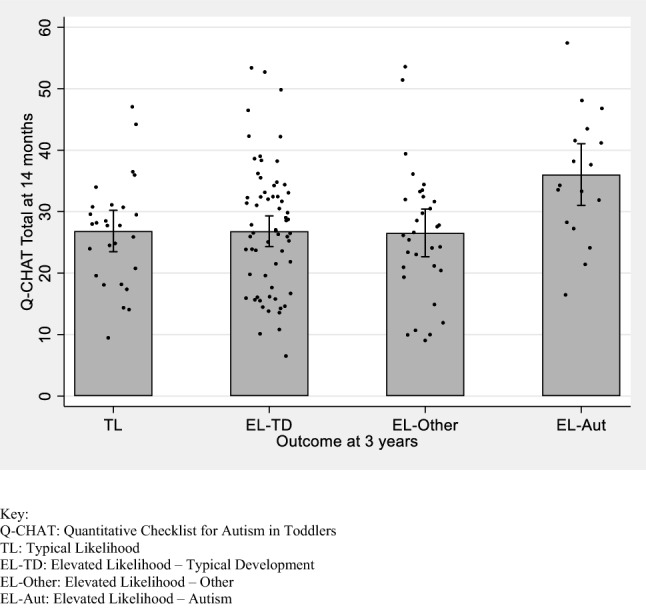
Mean Q-CHAT total score by outcome group

Q-CHAT total scores were negatively associated with concurrent Mullen ELC score (*r *= −.357, *p *< .001) but not with age at assessment (*r *= −.110, *p *= .196). There were significant between-group differences in Q-CHAT total scores at 14 months (*F*(3,136) = 4.403, *p *= .005, partial *η*^2^ = .089). Bonferroni post hoc tests showed that EL-Autism scores were higher than TL, EL-TD and EL-Other scores (*p *= .019, .005 and .010 respectively). There were no significant differences between the TL, EL-TD and EL-Other groups. Between-group differences were still significant when Mullen ELC scores were controlled for (*F*(3,135) = 2.850, *p *= .040, partial *η*^2^ = .060). Mean Q-CHAT total scores by outcome group are shown in Fig. 1. A multivariate ANOVA including all 25 Q-CHAT items showed 9 items with between-group differences: *Response to joint attention* scores were higher in the EL-Autism group than in the three other groups and five items (*Response to name*, *Pointing to request*, *Pointing to share interest*, *Offering comfort* and *Gestures*) had higher scores for the EL-Autism group than for both the TL and EL-TD groups. Full details for individual items are shown in the Supplementary Materials.

### AOSI Scores at 14 Months

**Fig. 2:**
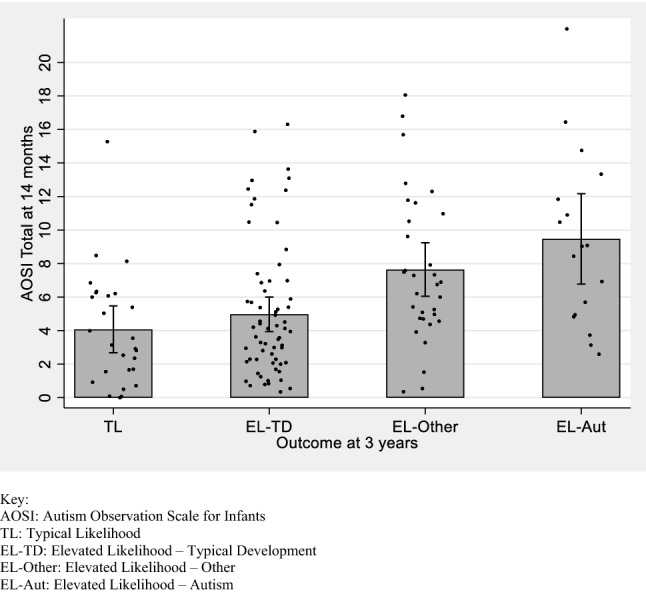
Mean AOSI total score by outcome group

AOSI total scores were negatively associated with concurrent Mullen ELC score (*r *= -.429, *p *< .001) but not with age at assessment (*r *= −.090, *p *= .289). There were significant between-group differences in AOSI total scores at 14 months (*F*(3,136) = 8.560, *p *< .001, partial *η*^2^ = .159). Bonferroni post hoc tests showed that both EL-Other and EL-Autism scores were higher than both TL and EL-TD scores (*p *= .009 and .025 for EL-Other and *p *< .001 and = .001 for EL-Autism respectively). There was no significant difference between EL-Other and EL-Autism scores (*p *= .912). Between-group differences remained significant when Mullen ELC scores were controlled for (*F*(3,135) = 4.089, *p *= .008, partial *η*^2^ = .083). Mean AOSI total scores by outcome group are shown in Fig. 2. A multivariate ANOVA including all individual AOSI items showed three items with between-group differences: *Respond to name* scores were higher in the EL-Autism group than in the TL and EL-TD groups, *Transitions* scores were higher in the EL-Autism group than in the EL-TD group and *Eye contact s*cores were higher in the EL-Other group than in the TL group. Full details for individual items are shown in the Supplementary Materials.

### Parental Concerns at 14 Months

Thirty-three parents (23.6%) expressed concerns about their child at 14 months. Three parents in the TL group (11.1%) and ten parents from each EL outcome group (representing 15.7%, 31.3% and 58.9% for the EL-TD, EL-Other and EL-Autism groups respectively) expressed concerns. There were no differences in concurrent Mullen ELC scores or ages at assessment between the group about whom parents expressed concerns and those about whom parents did not express concerns (*t *= 1.780, *p *= .077). Concerns were expressed about *Language development* (*n *= 5), *Stereotyped behaviours* (*n *= 2), *Motor skills* (*n *= 5), *General behaviour problems* (*n *= 20) and *Autism Spectrum Disorder* (*n *= 1). The numbers of parents from each group expressing concerns are shown in Table 2.

**Table 2:** Numbers of parents expressing concerns at 14 months

	TL^1^ (*n *= 27)	EL-TD^2^ (*n *= 64)	EL-Other^3^ (*n *= 32)	EL-Autism^4^ (*n *= 17)	All (*n *= 140)
No concern	24	54	22	7	107
Language development	0	2	2	1	5
Stereotyped behaviour	0	1	0	1	2
Motor skills	1	1	2	1	5
Behaviour	2	5	6	7	20
ASD	0	1	0	0	1
Total concerns (%)	3 (11.1%)	10 (15.7%)	10 (31.3%)	10 (58.9%)	33 (23.6%)

^1^Typical Likelihood

^2^Elevated Likelihood—typical development

^3^Elevated Likelihood—other

^4^Elevated Likelihood—autism

A Pearson *χ*^2^ test for the number of concerns showed significant between-group differences (*χ*^2^ = 17.34, *df *= 3, *p *= .001). There were significantly more concerns about children in the EL-Autism group than about those in the TL and EL-TD groups (*χ*^2^ = 11.41, *p *= .001 and *χ*^2^ = 13.48, *p *< .001 respectively) and marginally but not significantly more than in the EL-Other group (*χ*^2^ = 3.49, *p *= .062).

### Relationships Between Measures

**Fig. 3:**
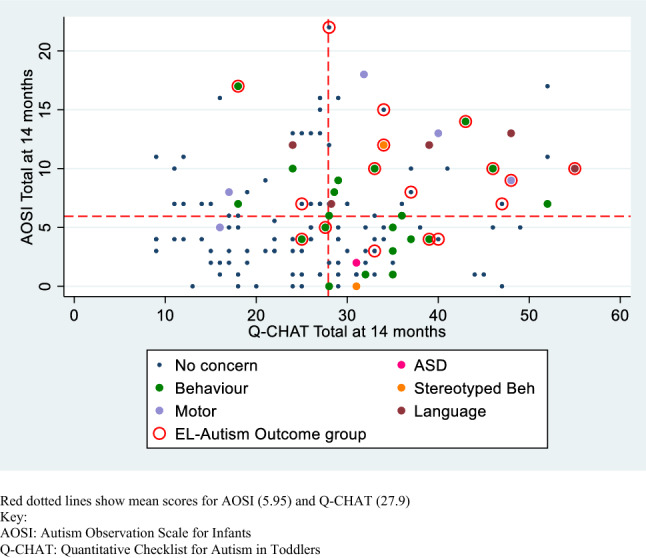
Scatterplot of Q-CHAT total × AOSI total score, showing domain of parental concern and EL-Autism group

There was no association between AOSI total and Q-CHAT total scores (*r *= .148, *p *= .081). These associations were also investigated within each outcome group, and none of these were significant. The results of these within-group tests are presented in the Supplementary Materials. Children about whom parents had concerns had higher Q-CHAT total scores (*t *= -3.48, *p *= .001, *d *= 0.69) and higher AOSI total scores (*t *= −2.47, *p *= .015, *d *= 0.48) than those of parents without concerns. Figure 3 shows a scatterplot of AOSI total and Q-CHAT total scores, with the sample means for these two measures, domain of parental concern and EL-Autism group indicated. To investigate whether looking at all three measures in combination was informative, we considered three criteria: (1) scoring above the mean for the Q-CHAT; (2) scoring above the mean for the AOSI; (3) the presence of parental concern. All 17 children in the EL-Autism group met at least one of these criteria. Twelve of these children met at least two criteria. Across the other outcome groups the numbers and percentages of children meeting at least one and at least two criteria in each group was as follows: TL: 18 (66.7%) & 6 (22.2%); EL-TD: 39 (60.9%) & 16 (25%); EL-Other: 27 (84.4%) & 14 (43.8%).

Scores from individual items relating to the same or similar concepts from the AOSI and the Q-CHAT were compared. None of the eleven comparisons tested showed significant associations (with significance set to *p *< .01 to account for multiple comparisons). Details of these comparisons are shown in the Supplementary Materials.

### Intervention Trial Supplementary Analysis

For Q-CHAT total and AOSI total scores we conducted univariate ANOVAs to test for main effects of RCT participation or receiving intervention, with binary variables representing participation in the RCT and receiving intervention as the independent variables. We then repeated the univariate ANOVAs with diagnostic outcome group as the independent variable and the RCT and intervention variables as covariates. There were no main effects of either RCT or intervention variable on Q-CHAT or AOSI total scores (all *F *< 1.8). Controlling for RCT participation and receiving intervention, between-group differences remained significant for both Q-CHAT and AOSI total scores (*F*(3,134) = 4.447, *p *= .005, partial *η*^2^ = .091 and *F*(3,134) = 8.366, *p *< .001, partial *η*^2^ = .158 respectively). For the expression of parent concerns, we conducted Pearson *χ*^2^ tests for the number of concerns across RCT participation and intervention groups. In both cases there were no significant differences (RCT: *χ*^2^ = 2.07, *df *= 1, *p *= .150; intervention: *χ*^2^ = 1.36, *df *= 1, *p *= .244). Follow-up analyses were done by first excluding participants who took part in the RCT and then those who received intervention. In each case the pattern of results reported for the main findings above were the same (i.e. there were significantly more concerns reported by parents of children later diagnosed with autism than those in the TL and EL-TD groups, but no significant differences between the EL-Autism and EL-Other groups, despite relatively large differences in proportions of expressed concerns—not in RCT: 54.5% vs 23.5%; no intervention: 50.0% vs 30.4%). Full details are reported in the Supplementary Materials.

## Discussion

This is the first study to investigate the relative utility of three different concurrent sources of information about later autism diagnosis in children in the early part of their second year of life. Q-CHAT total scores at 14 months were significantly higher for the children later diagnosed with autism, AOSI total scores distinguished children with autism from those who were developing typically, but not from those with apparent non-autism developmental difficulties. Parents of children subsequently diagnosed with autism were more likely to report concerns about their child’s development than parents of children with no apparent later developmental difficulties and almost twice as likely to report concerns compared to parents of children who subsequently had developmental difficulties but not autism, although this difference was not statistically significant due to the relatively modest sample. The expression of parental concerns at 14 months was associated both with higher Q-CHAT and AOSI total scores.

Individual items from the two instruments that were higher in the group later diagnosed with autism were mostly related to social communication and interaction skills, particularly *Response to joint attention, Gestures*, *Pointing to request*, *Pointing to share interest*, *Offering comfort* and *Response to name* from the Q-CHAT and *Respond to name* from the AOSI. In contrast, the concerns expressed by seven of the ten parents whose children were subsequently diagnosed with autism were about broad behavioural issues rather than about aspects of social interaction, language or communication. Examples of these behavioural concerns include “Headbutting when upset, or if you take him away from something he wants to do—can hurt himself too”, “…definitely going through the terrible two’s—throws things all the time and has no sense of danger” and “… gets very upset when he can’t see his mum”. Only five of all parents expressed concerns about unusual or delayed language and just two were concerned about unusual, repetitive or stereotyped behaviour. The one child whose parent was explicitly concerned about autism subsequently appeared to be developing typically. However, whilst parents did not *explicitly* identify concerns about social communication and interaction it does appear that they were able *implicitly* to recognise differences in their children’s early social communication skills when endorsing scores on relevant items on the Q-CHAT.

Our findings regarding the Q-CHAT broadly correspond to those reported by Sacrey et al. (2018) in relation to their use of the 26-item APSI parent-report questionnaire at 12 months. They found that APSI total scores were higher in children with ASD outcomes in their EL sample compared to both EL non-ASD and TL groups, and all seven individual items that exclusively distinguished the ASD group from the other two groups were reflective of social communication and interaction skills (i.e. *Respond to name*, *Anticipatory social response*, *Eye contact*, *Reciprocal social smile*, *Reactivity*, *Cuddle with you*, and *Share interests with others*). Furthermore, the six items from our study that distinguished children subsequently diagnosed with autism from those in the TL and EL-TD groups all appear in the short version of the Q-CHAT presented by Allison, Auyeung and Baron-Cohen (2012) that includes the ten items found to have the highest positive predictive values. The *Respond to name* item was the strongest indicator of autism outcome from the AOSI in our study—a finding that has been reported in other EL studies where the AOSI has been used between 12 and 18 months (e.g. Gammer et al. 2015; Sacrey et al. 2018) and which confirms the importance of measuring this behaviour in toddlers for whom autism is being considered. Overall, our findings from the AOSI showing differences in early emerging autism symptomatology at 14 months confirm findings from previous studies about early behavioural manifestations of autism being detectable from soon after the child’s first birthday using semi-structured examiner-led instruments (e.g. Brian et al. 2008; Elison et al. 2014; Gammer et al. 2015; Ozonoff et al. 2010).

With regard to the expression of parental concerns, our findings are not directly comparable with those reported by Sacrey et al. (2015) in that they had a much larger sample of children later diagnosed with autism, their data were based on a semi-structured interview with pre-determined aspects of development and concerns about more than one area were potentially counted for each participant at each time point. However, there is a partial concordance with our findings: Sacrey et al. (2015) report that at both 12 and 15 months the overall number of concerns were higher for the children later diagnosed with ASD than in both of the non-ASD comparison groups, and around 30% of parents of children in the ASD group expressed concerns about general behavioural problems. In contrast to our findings Sacrey et al. (2015) report that both sensory and communication issues distinguished the ASD outcome group from the non-ASD groups at every time point between 6 and 24 months.

Measures pertaining to similar constructs ascertained via different methods—direct interactive assessment with a child versus parental interview, for example—often have poor agreement, even when two measures have identical items (e.g. Sacrey et al. 2018). Even though instruments such as the AOSI and the ADOS are designed to elicit behaviours known to be symptomatic of autism, children who display these in their everyday behaviour may not exhibit them within a relatively brief assessment. Their parents, however, may be fully aware of, and report, the presence of these behaviours, such as mannerisms, echolalic speech, unusual interests, and so on. Alternatively, in relation to specific aspects of early social communication skills such as responding to joint attention, many parents may not be aware of this as a behaviour of interest and therefore may not report the presence or absence of this behaviour reliably, yet within the context of an examiner-administered semi-structured assessment this may be a target behaviour with a relatively observable response. It is not unexpected, therefore, that items from our two instruments relating to identical or overlapping concepts did not correlate, and that the three measures reported here showed differences in terms of their ability to discriminate later autism diagnostic status.

### Limitations

The primary limitation of this study is that data are drawn from a prospective longitudinal study of young children at elevated likelihood of autism diagnosis, and therefore the findings may not generalise to a community-based context (Sacrey et al. 2017). One consequence of the study design is that the majority of parents have an older child with autism, which is likely to influence responses to questions about their child’s early development as well as their perception of and attitude towards putative problem behaviours and difficulties in their young child. Even though parents who have older children with autism are inevitably better informed about the emerging signs of autism than most parents of young children, it is not clear to what extent or in what direction the reporting of concerns will be affected—they may be ‘hypervigilant’ to every potential manifestation associated with autism, or their ‘calibration’ of behaviours in comparison to potential typical development may be such that they have a tendency to under-report autistic-like behavioural symptoms. A second consequence of the study design is that even the children assigned a diagnosis of autism at 3 years were relatively low in terms of their autism symptomatology, particularly in relation to their scores on the ADOS: the EL-Autism group ADOS-2 Calibrated Severity Score mean of 3.8 is lower than the lower cut-off score of 4 that corresponds to an ADOS-2 classification of autism spectrum. In spite of this relatively low severity, however, the instrument scores and expression of concerns still distinguish the children with autism from their non-autistic counterparts to some extent. We might assume that were it possible to recruit a sample of children with more profound levels of autism severity, these measures would distinguish children with autism more strongly. A final limitation relating to the study design is the relatively small sample, which contains just 17 children diagnosed with autism.

Our additional analyses relating to the children who took part in the early intervention trial described in Green et al. (2017) demonstrate that neither participation in the trial nor being in receipt of the intervention had a significant impact on the pattern of findings relating to the sample as a whole. Given that the active intervention phase of the trial ended shortly before the children were assessed at 14 months, it is not unlikely that the scores on either the AOSI or the Q-CHAT, or the expression of concerns about a child’s development, would have been influenced to some extent by participation in the intervention programme. Indeed, Green et al. (2017) report non-significant benefits in AOSI scores at this visit for the children in the intervention arm of the trial, although no claims for effects relating to longer term diagnostic outcome are made. It is beyond the scope of this report to further analyse potential treatment effects relating to the trial.

We do not have alternative measures of either parent-reported or examiner-observed autistic symptomatology at 14 months (the M-CHAT or the Toddler Module of the ADOS, for example) with which to make direct comparisons with our findings from the Q-CHAT and the AOSI. The measures investigated here show high levels of variance, so in conjunction with the highly heterogenous nature of autism, these findings can only be interpreted at the group level and are not readily interpretable for individual children. Professionals considering the likelihood of autism diagnosis for a young child should be cautious when presented with information from a single source, whether that be a semi-structured assessment, parental questionnaire or the expression of parental concerns.

## Electronic supplementary material

Below is the link to the electronic supplementary material.

Supplementary material 1 (DOCX 31 kb)

## Footnotes

1 We are using the term ‘elevated likelihood’ in place of the more commonly used term ‘high risk’ (HR) in response to parental preferences reported in Fletcher-Watson et al. (2017).
