# Supplementary material for: Comparison of Parent Questionnaires, Examiner-Led Assessment and Parents’ Concerns at 14 Months of Age as Indicators of Later Diagnosis of Autism
Source: J Autism Dev Disord. 2019 Dec 16;51(3):804–13. doi: 10.1007/s10803-019-04335-z (PMC7954743; doi:10.1007/s10803-019-04335-z)
Supplement: Supplementary file 1 — Supplementary material 1 (DOCX 31 kb) [file 10803_2019_4335_MOESM1_ESM.docx]

Diagnostic status of participants’ older siblings

For all 113 children with an older sibling with a community clinical diagnosis of ASD (hereafter probands) parents completed the Development and Well-Being Assessment (DAWBA: Goodman, Ford, Richards, Gatward, & Meltzer, 2000) and/or the Social Communication Questionnaire (SCQ: Rutter, Bailey, & Lord, 2003). Eighty-nine probands met criteria on both the DAWBA and SCQ. Seven children scored below threshold on the SCQ and one was missing the SCQ, but no exclusions were made due to meeting threshold on the DAWBA and expert opinion. For 16 probands, confirmation of local clinical diagnosis was only available via the SCQ. Screening for possible ASD in the older siblings of the typical likelihood (TL) infants was undertaken using the SCQ, with no child scoring above the instrument cut-off for ASD (>15). For one TL child the SCQ was missing. Medical history review confirmed a lack of ASD within first-degree relatives.

References

Goodman, R., Ford, T., Richards, H., Gatward, R., & Meltzer, H. (2000). The Development and Well-Being Assessment: description and initial validation of an integrated assessment of child and adolescent psychopathology. *Journal of Child Psychology & Psychiatry, 41*(5), 645-655.

Rutter, M., Bailey, A., & Lord, C. (2003). *The Social Communication Questionnaire*. Los Angeles, CA: Western Psychological Services.

SM Table 1.

Individual Q-CHAT* Items and contribution to between-group differences. Items with p<.05 shown in bold

| **Item** | ***F*(3,132)** | ***p*** | **Partial *η*^2^** | **Post-hoc differences** | |
| --- | --- | --- | --- | --- | --- |
| **Response to Name** | **3.06** | **.031** | **0.065** | **EL-Aut > TL & EL-TD (*p*=.029 & *p*=.048)** | |
| Eye Contact | 1.54 | .208 | 0.034 |  |  |
| Lining Up Objects | 1.01 | .130 | 0.042 |  | |
| **Intelligibility** | **2.72** | **.047** | **0.058** | NB – no individual comparisons significant at p<.05 | |
| **Pointing to Request** | **5.09** | **.002** | **0.104** | **EL-Aut > TL & EL-TD (*p*=.002 & *p*=.012)** | |
| **Pointing to Share Interest** | **4.44** | **.005** | **0.092** | **EL-Aut > TL & EL-TD (*p*=.003 & *p*=.019)** | |
| Staring at Spinning Objects | 1.22 | .306 | 0.027 |  |  |
| Number of Words | 2.58 | .056 | 0.055 |  | |
| **Pretend Play** | **3.29** | **.023** | **0.070** | **EL-Aut > TL (*p*=.021)** | |
| **Response to Joint Attention** | **4.41** | **.005** | **0.091** | **EL-Aut > TL, EL-TD & EL-Other (*p*=.007, *p*=.032 & *p*=.009)** | |
| **Unusual Sensory Interests** | **3.42** | **.019** | **0.072** | **TL > EL-TD & EL-Other (*p*=.039 & *p*=.027)** | |
| Use of Other’s Hand as a Tool | 1.53 | .209 | 0.034 |  |  |
| Tiptoe Walking | 2.21 | .090 | 0.048 |  | |
| Flexibility | 0.64 | .594 | 0.014 |  |  |
| **Offering Comfort** | **3.29** | **.023** | **0.070** | **EL-Aut > TL & EL-TD (*p*=.022 & *p*=.037)** | |
| Repetitive Behaviour | 1.52 | .211 | 0.033 |  |  |
| Typicality of First Words | 0.98 | .404 | 0.022 |  |  |
| **Echolalia** | **3.44** | **.019** | **0.072** | **EL-Other > EL-Aut (*p*=.047)** | |
| **Gestures** | **5.16** | **.002** | **0.105** | **EL-Aut > TL & EL-TD (*p*=.001 & *p*=.014)** | |
| Mannerisms | 0.27 | .850 | 0.006 |  |  |
| Social Referencing | 1.04 | .376 | 0.023 |  |  |
| Intense Interest in Objects | 1.18 | .321 | 0.026 |  |  |
| Repetitive Use of Objects | 0.36 | .781 | 0.008 |  |  |
| Oversensitivity to Noise | 1.16 | .328 | 0.026 |  |  |
| Staring at Nothing | 0.70 | .553 | 0.016 |  |  |

* Quantitative Checklist for Autism in Toddlers

SM Table 2.

Individual AOSI* Items and contribution to between-group differences. Items with p<.05 shown in bold

| **Item** | ***F*(3,136)** | ***p*** | **Partial *η*^2^** | **Post-hoc differences** | |
| --- | --- | --- | --- | --- | --- |
| Visual Tracking | 1.58 | .197 | 0.034 |  |  |
| Disengagement of Attention | 0.61 | .609 | 0.013 |  |  |
| **Respond to Name** | **8.12** | **<.001** | **0.152** | **EL-Aut > TL, EL-TD & EL-Other (*p*=.001, <.001 & =.049, resp.)** | |
| Differential Responses to Facial Expressions | 1.27 | .288 | 0.027 |  |  |
| Anticipatory Responses (to Peekaboo) | 1.34 | .264 | 0.029 |  |  |
| Imitation of Actions | 0.47 | .705 | 0.010 |  |  |
| Social Babbling | 2.19 | .092 | 0.046 |  |  |
| **Eye Contact** | **3.83** | **<.001** | **0.078** | **EL-Other > TL (*p*=.011)** | |
| Reciprocal Social Smile | 2.29 | .081 | 0.048 |  |  |
| Coordination of Eye Gaze & Action | 2.17 | .094 | 0.046 |  |  |
| Reactivity | 1.54 | .208 | 0.033 |  |  |
| Social Interest & Shared Affect | 2.06 | .108 | 0.044 |  |  |
| **Transitions** | **3.66** | **.014** | **0.075** | **EL-Aut > EL-TD (*p*=.009)** | |
| Motor Control & Behaviour | 1.32 | .272 | 0.028 |  |  |
| Atypical Motor Behaviours | 2.57 | .057 | 0.054 |  |  |
| Atypical Sensory Behaviours | 2.04 | .112 | 0.043 |  |  |

*Autism Observation Scale for Infants

SM Table 3

**Results of within-group correlations between Q-CHAT^1^ total and AOSI^2^ total scores**

| **Group** | **Pearson’s *r*** | ***p*** | ***n*** |
| --- | --- | --- | --- |
| Typical Likelihood | -.039 | .845 | 27 |
| Elevated Likelihood – Typically Developing | .139 | .274 | 64 |
| Elevated Likelihood – Other | .157 | .390 | 32 |
| Elevated Likelihood – Autism | -.165 | .528 | 17 |

Key:

1 Quantitative Checklist for Autism in Toddlers

2 Autism Observation Scale for Infants

SM Table 4

**Correlations between individual items from the Q-CHAT and AOSI measuring similar constructs**

(All *n*=136)

| **Q-CHAT^1^ item** | **AOSI^2^ item** | **Spearman’s rho** | ***p*** |
| --- | --- | --- | --- |
| Response to name | Orientation to name | .089 | .303 |
| Eye contact | Eye contact | -.083 | .335 |
| Flexibility | Transitions | .113 | .190 |
| Response to joint attention | Disengagement of attention | -.042 | .630 |
| Social referencing | Social referencing | .010 | .909 |
| Staring at spinning objects | Atypical sensory behaviours | -.033 | .704 |
| Unusual sensory interests | Atypical sensory behaviours | .017 | .848 |
| Oversensitivity to noise | Atypical sensory behaviours | .080 | .353 |
| Staring at nothing | Atypical sensory behaviours | .197 | .022 |
| Mannerisms | Atypical motor behaviours | -.004 | .963 |
| Repetitive behaviour | Atypical motor behaviours | -.005 | .957 |

Key:

1 Quantitative Checklist for Autism in Toddlers

2 Autism Observation Scale for Infants

Intervention trial supplementary analyses

SM Table 5

**Numbers of children participating in** **Randomised Control Trial (RCT)**

|  |  |  |  |  |
| --- | --- | --- | --- | --- |
|  |  | In RCT | Not in RCT | Total |
| Outcome at 3 yrs | TL^1^ | 0 | 27 | 27 |
|  | EL-TD^2^ | 32 | 32 | 64 |
|  | EL-Other^3^ | 15 | 17 | 32 |
|  | EL-Autism^4^ | 6 | 11 | 17 |
|  | Total | 53 | 87 | 140 |

Key:

^1^ Typical Likelihood

^2^ Elevated Likelihood – Typical Development

^3^ Elevated Likelihood – Other

^4^ Elevated Likelihood – Autism

SM Table 6

**Numbers and percentages of concerns expressed at 14 months by participation in Randomised Control Trial (RCT)**

|  | TL^1^ | EL-TD^2^ | | EL-Other^3^ | | EL-Autism^4^ | | Total | |
| --- | --- | --- | --- | --- | --- | --- | --- | --- | --- |
| RCT participation | Not RCT | In RCT | Not RCT | In RCT | Not RCT | In RCT | Not RCT | In RCT | Not RCT |
| No Concern | 24 | 26 | 28 | 9 | 13 | 2 | 5 | 37 | 70 |
| Concern | 3 | 6 | 4 | 6 | 4 | 4 | 6 | 16 | 17 |
| Percentage concerns | 11.1 | 18.3 | 12.5 | 40.0 | 23.5 | 66.7 | 54.5 | 30.2 | 19.5 |

Key:

1 Typical Likelihood

^2^ Elevated Likelihood – Typical Development

^3^ Elevated Likelihood – Other

^4^ Elevated Likelihood – Autism

SM Table 7

**Numbers of children in Randomised Control Trial (RCT) Intervention group or case series**

|  |  |  |  |  |
| --- | --- | --- | --- | --- |
|  |  | Intervention | No intervention | Total |
| Outcome at 3 yrs | TL^1^ | 0 | 27 | 27 |
|  | EL-TD^2^ | 18 | 46 | 64 |
|  | EL-Other^3^ | 9 | 23 | 32 |
|  | EL-Autism^4^ | 5 | 12 | 17 |
|  | Total | 32 | 108 | 140 |

Key:

1 Typical Likelihood

^2^ Elevated Likelihood – Typical Development

^3^ Elevated Likelihood – Other

^4^ Elevated Likelihood – Autism

SM Table 8

**Numbers and percentages of concerns expressed at 14 months by participation in Randomised Control Trial (RCT) / case series intervention**

|  | TL^1^ | EL-TD^2^ | | EL-Other^3^ | | EL-Autism^4^ | | Total | |
| --- | --- | --- | --- | --- | --- | --- | --- | --- | --- |
| Intervention | No | Yes | No | Yes | No | Yes | No | Yes | No |
| No Concern | 24 | 15 | 39 | 6 | 16 | 1 | 6 | 22 | 85 |
| Concern | 3 | 3 | 7 | 3 | 7 | 4 | 6 | 10 | 23 |
| Percentage concerns | 11.1 | 16.7 | 15.2 | 33.3 | 30.4 | 80.0 | 50.0 | 30.2 | 21.3 |

Key:

^1^ Typical Likelihood

^2^ Elevated Likelihood – Typical Development

^3^ Elevated Likelihood – Other

^4^ Elevated Likelihood – Autism
